# 

**Published:** 1815-11

**Authors:** 


### LECTURES.

Mr. Carmichael will deliver a Course of Clinical Lectures on Syphilis, and the Diseases which have been confounded with it at the Lock Hospital, Dublin, on Mondays and Thursdays; to commence the second Monday in January, 1816, at half-past eleven o'clock. The principal object of these Lectures will be to point out the distinctive characters of the several classes and species of Venereal Diseases.

Mr. Carpue commenced his Lectures on Anatomy, at his Theatre, No. 50, Dean-street, Soho, on Monday, the second of October, 1815.

Mr. Singer will commence a Course of Lectures on Electricity and Electro-Chemical Science, on Monday, the 6th of November, at his Lecture Room, 3, Princes-street, Cavendish-square.

---

### BOOKS IN THE PRESS.

The Student's Journal, arranged, printed, and ruled for receiving an account of every day's employment for the space of one year, and designed for the use of professional students, scientific and literary men, and readers in general.

In one neat pocket volume, the Practice of Physic by the late Dr. William Cullen; containing all the modern improvements in medicine, and a collection of the most useful Formulæ. To which will be added, a new and correct Translation of the New London Pharmacopœia, with explanatory Notes, Indexes, &c.

---

The Diseases of the present month having varied but little from the preceding, we shall, on account of our great press of matter, defer our remark on particular cases to the next month.

### MONTHLY CATALOGUE OF MEDICAL BOOKS.

Report, together with the Minutes of Evidence, and an Appendix of Papers from the Committee appointed to consider of Provision being made for the better regulation of Madhouses in England, (ordered by the House of Commons to be printed, 11th July, 1815). Each subject of evidence arranged under its distinct head, by J. B. Sharpe, Member of the Royal College of Surgeons. 8vo. Baldwin and Co.

A System of the Anatomy of the Human Body; illustrated by upwards of two hundred tables, taken partly from the most celebrated authors, and partly from Nature. By Andrew Fyfe. Third edition, considerably enlarged and improved. 3 vols. 4to. Anderson.

Albinus's Plates of the Human Muscles, carefully reduced from the original copy; accompanied with concise explanations, for the use of students. 12mo. Cox and Son.

Cheselden's Plates of the Human Bones, carefully reduced from the original copy; accompanied with concise explanations, for the use of students. 12mo. Cox and Son.

Smellie's Anatomical Plates of Midwifery, selected and reduced from the large folio copy; with concise explanations. 18mo. E. Cox and Son.

Elements of Conchology, according to the Linnean System; illustrated by 28 plates, drawn from Nature. By the Rev. E. J. Burrow, A. M. F.L.S. Mem. Geol. Soc. 8vo. Underwood.

Appendix to a Catalogue of Books in Anatomy, Surgery, Medicine, Midwifery, Chemistry, Botany, &c. new and second hand for 1815-16. Sold by John Anderson, Medical Bookseller, 40, West Smithfield, London.

The Pharmacopoeia of the Royal College of Physicians of London, 1809; translated into English, with notes, by Dr. Powell. The third edition, 8vo. Longman and Co.

The New London Pharmacopœia, correctly translated from the last Latin edition, and rendered more convenient for the purposes of practice, by the addition of the properties, doses, &c. of each preparation; together with appropriate and copious Tables and Indexes. By R. J. Thornton, M.D. Member of the Royal College of Physicians, London. 18mo. Cox and Son.

Pharmacopœia Officinalis Britannica, being a new and correct Translation of the late edition of the London Pharmacopœia; with which are incorporated, in alphabetical order, all the Formulæ of the Edinburgh and Dublin Colleges; together with notes explanatory of the different processes. The whole corrected agreeably to the late alterations of the New London Pharmacopœia. By Richard Stocker, Apothecary to Guy's Hospital. 8vo. Cox and Son.

A Translation of the corrected edition of the Pharmacopoeia Collegii Regalis, Medicorum Londinensis; published in July 1815, with notes. By a London Physician. 8vo. Highley and Son.

The Elements of Experimental Chemistry. By William Henry, M.D. F.R.S. &c. &c. &c. The seventh edition, greatly enlarged, and illustrated with nine plates, engraved by Lowry. 2 vols. Baldwin and Co.

Cases of Diseased Bladder and Testicle; illustrated with seventy-one etchings. By William Wadd. 4to. Callow.

---

### *NOTICES TO CORRESPONDENTS.*

*A GENERAL INDEX to the Thirty-Three Volumes of this Journal is now in forwardness; and, as the Proprietors intend to annex a* List of Subscribers' Names *thereto, and a very few more than are subscribed for will be printed, they solicit the Favor of Names, as soon as possible.*

*Our Friends will please to be particular in the Address of their Communications, which should be thus,* "To the Editors of the London Medical and Physical Journal, at Mr. Souter's, No. 1, Paternoster-Row; or at Mr. Adlard's, 23, Bartholomew-Close."

*Mr. Carmichael's Paper shall appear as soon as we can procure an Engraving sufficiently illustrative of the diseased Appearances.*

*We have also been favoured with Communications from Drs.* Meyler; Ronalds; *and* R. N. Starr, W. J. Crowfoot, J. Wayte, *Esquires;* Medicus; *and several Letters from* Apprentices; *to all of which we shall pay due attention.*

*We wish such gentlemen as are unwilling to give either their names or residence, would add a signature of some kind, that we may be enabled to notice our receipt of their papers.*

